# Cell therapy for spinal cord injury by using human iPSC-derived region-specific neural progenitor cells

**DOI:** 10.1186/s13041-020-00662-w

**Published:** 2020-09-03

**Authors:** Keita Kajikawa, Kent Imaizumi, Munehisa Shinozaki, Shinsuke Shibata, Tomoko Shindo, Takahiro Kitagawa, Reo Shibata, Yasuhiro Kamata, Kota Kojima, Narihito Nagoshi, Morio Matsumoto, Masaya Nakamura, Hideyuki Okano

**Affiliations:** 1 Department of Physiology, Keio University School of Medicine, 35 Shinanomachi, Shinjuku-ku, Tokyo, 160-8582 Japan; 2 Department of Orthopaedic Surgery, Keio University School of Medicine, 35 Shinanomachi, Shinjuku-ku, Tokyo, 160-8582 Japan; 3 Electron Microscope Laboratory, Keio University School of Medicine, 35 Shinanomachi, Shinjuku-ku, Tokyo, 160-8582 Japan

**Keywords:** Spinal cord injury, Stem cell therapy, Region-specific neural progenitors

## Abstract

The transplantation of neural progenitor cells (NPCs) derived from human induced pluripotent stem cells (iPSCs) has beneficial effects on spinal cord injury (SCI). However, while there are many subtypes of NPCs with different regional identities, the subtype of iPSC-derived NPCs that is most appropriate for cell therapy for SCI has not been identified. Here, we generated forebrain- and spinal cord-type NPCs from human iPSCs and grafted them onto the injured spinal cord in mice. These two types of NPCs retained their regional identities after transplantation and exhibited different graft-host interconnection properties. NPCs with spinal cord regional identity but not those with forebrain identity resulted in functional improvement in SCI mice, especially in those with mild-to-moderate lesions. This study highlights the importance of the regional identity of human iPSC-derived NPCs used in cell therapy for SCI.

## Introduction

Spinal cord injury (SCI) is a devastating and incurable disease, and basic treatment has not yet been developed. While many therapeutic options have been studied, neural progenitor cell (NPC) transplantation is one of the most promising choices for SCI treatment. It has been suggested that transplanted neural cells facilitate the regeneration of injured spinal cords by reconstructing local neural circuits, providing a scaffold for axonal growth, and supplying trophic support [1]. We and other groups have indeed demonstrated the efficacy of the transplantation of NPCs derived from induced pluripotent stem cells (iPSCs) [2–7], and the first in-human clinical trial of iPSC-derived NPCs for SCI patients is expected to be conducted within a few years [8].

Before iPSC technology was established, undifferentiated cells derived from fetal tissues were intensively studied as the cell source for SCI treatment [9–13]. Indeed, many studies suggested that the functional improvement of SCI model animals was achieved by the transplantation of fetal cells with multiple origins, such as the spinal cord [9, 10], the forebrain [11, 12], and the peripheral tissues [13]. That said, it has been recently demonstrated that the original identity of transplanted fetal NPCs is important for tissue regeneration and functional restoration [14, 15]. In these studies, fetal spinal cord NPCs resulted in better effects than NPCs with other original identities. Although the underlying mechanisms remain to be understood, these studies suggest that NPCs with a spinal cord identity may prove to be a more desirable cell source for SCI treatment.

However, in SCI cell therapy using iPSC-derived NPCs, verification of the regional identity has not been extensively performed. In particular, it has not yet been determined which regional specificity is suited for iPSC-derived NPCs for restoring the motor function of SCI model animals. The characteristics of iPSC-derived NPCs are not the same as those of fetal NPCs in several aspects, including the differentiation capacity, gene expression pattern, and epigenetic status [16]; thus, it is essential to clarify the significance of iPSC-derived NPC regional identity for spinal cord regeneration. Fortunately, techniques for controlling the regional identity of iPSC-derived NPCs has been actively developed in recent years. Several studies have reported that regional patterning could be achieved by modulating the gradients of morphogens, such as Wnt and retinoic acid (RA), during neural induction of iPSCs [17–20]. Although these regionalized NPCs have been successfully used for disease modeling and drug screening [20–24], the regional specification of iPSC-derived NPCs used for regenerative medicine to treat SCI has been poorly investigated, particularly from the point-of-view of functional recovery.

In this study, we generated regionalized NPCs with a forebrain or spinal cord identity from iPSCs and examined the effect of these NPCs on spinal cord regeneration. Both forebrain- and spinal cord-type NPCs retained their positional identities after transplantation, but only the spinal cord-type NPCs interconnected with host neural circuits. Spinal cord-type but not forebrain-type NPCs resulted in motor function recovery in SCI mice. Furthermore, a stratification approach revealed that host responsiveness to spinal cord-type NPC transplantation is linked to several histological characteristics. Our work underscores the importance of the regional identity of iPSC-derived NPCs for cell therapy and provides practical instructions to develop complete treatments for SCI.

## Results

### Generation of human iPSC-derived NPCs with specific regional identities

**Fig. 1:**
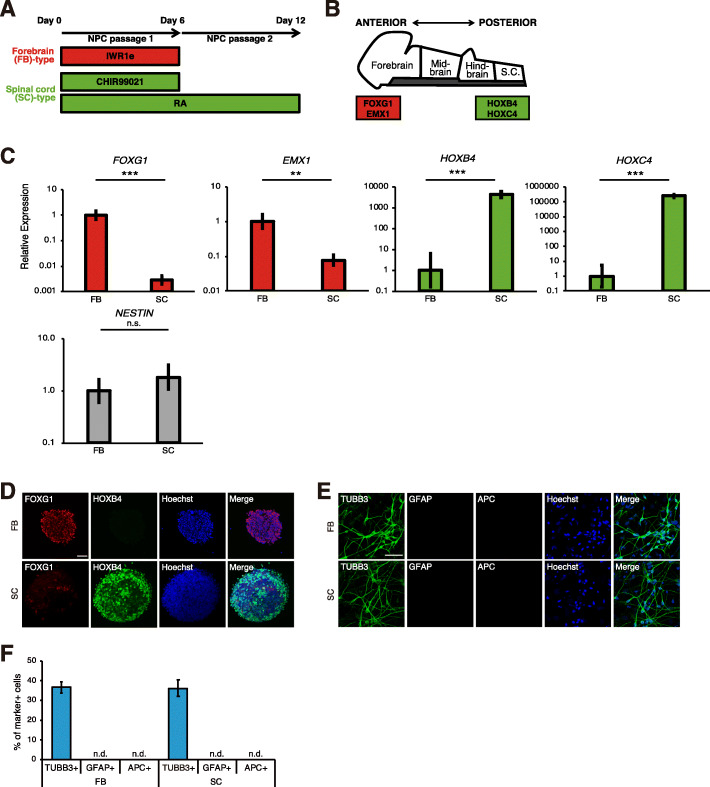
Regionalization of human iPSC-derived NPCs. **a** Overview of the culture protocol. Patterning factors (IWR1e, CHIR99021, and RA) were added during NPC induction. **b** Expression of regional markers in the neural tube in vivo. **c** qRT-PCR analysis of iPSC-derived NPCs to detect regional marker expression (*n* = 3–6 independent experiments; mean ± SD; ***p < 0.001, **p < 0.01; n.s., not significant; Student’s *t* test). **d** Immunocytochemical analysis of iPSC-derived NPCs to detect regional markers. Scale bar, 50 μm. **e** Immunocytochemical analysis of NPC-derived neurons, astrocytes, and oligodendrocytes. Scale bar, 50 μm. **f** Quantification of the number of NPC-derived neurons, astrocytes, and oligodendrocytes (*n* = 3 independent experiments; mean ± SD; n.d., not detected). iPSC-derived NPCs preferentially differentiated into neurons rather than astrocytes or oligodendrocytes

To generate region-specific NPCs from human iPSCs, we first regulated Wnt and RA signaling during neural induction as previously described [20] (Fig. 1a). We used the Axin2 stabilizer IWR1e as a Wnt inhibitor [25] and the GSK3β inhibitor CHIR99021 as a Wnt activator [26]. We hypothesized that Wnt-inhibited and Wnt/RA-activated cells have the forebrain-type (FB-type) and the spinal cord-type (SC-type) identities, respectively. As expected, Wnt-inhibited (FB-type) cells showed high expression of the forebrain marker *FOXG1* and *EMX1*, and the spinal cord marker *HOXB4* and *HOXC4* were upregulated in Wnt/RA-activated (SC-type) cells, whereas the NPC marker *NESTIN* was not affected by these signaling modulators (Fig. 1b, c, S1A). These results were also confirmed by immunocytochemical analysis of the FOXG1 and HOXB4 proteins (Fig. 1d). We also compared the proliferative activity between FB- and SC-type NPCs by Ki67 immunostaining, and there were no significant differences (Fig. S1B, C). In addition, trypan blue staining revealed that both types of NPCs had a high cell viability (> 90%) (Fig. S1D). These results indicate that controlling Wnt and RA signaling enables the generation of FB- and SC-type NPCs, respectively, without influencing the differentiation, proliferation, and viability of the NPCs.

Next, we assessed the differentiation capacity of the regionalized NPCs. The differentiated cells were exclusively TUBB3-positive neurons, and we could not detect glial cells, including GFAP-positive astrocytes or APC-positive oligodendrocytes (Fig. 1e, f). There were no differences in the efficiency of neuronal production between the two types of NPCs. These results imply that FB- and SC-type NPCs have similar neural differentiation characteristics. In addition, we found that the TUBB3-negative cells were mostly immunopositive for the proliferative marker Ki67 and the neural stem/progenitor cell marker SOX1, indicative of residual neural progenitors (Fig. S1E, F). We detected the spinal cord interneuron marker ISL1/2- and BRN3A-positive neurons in the SC-type culture, while we did not detect motor neuron marker (HB9 and ChAT) expression, suggesting that SC-type NPCs mainly differentiated into spinal cord interneurons (Fig. S1G, H).

### Preservation of the regional identity and in vivo differentiation of iPSC-derived NPC grafts in injured spinal cords

**Fig. 2:**
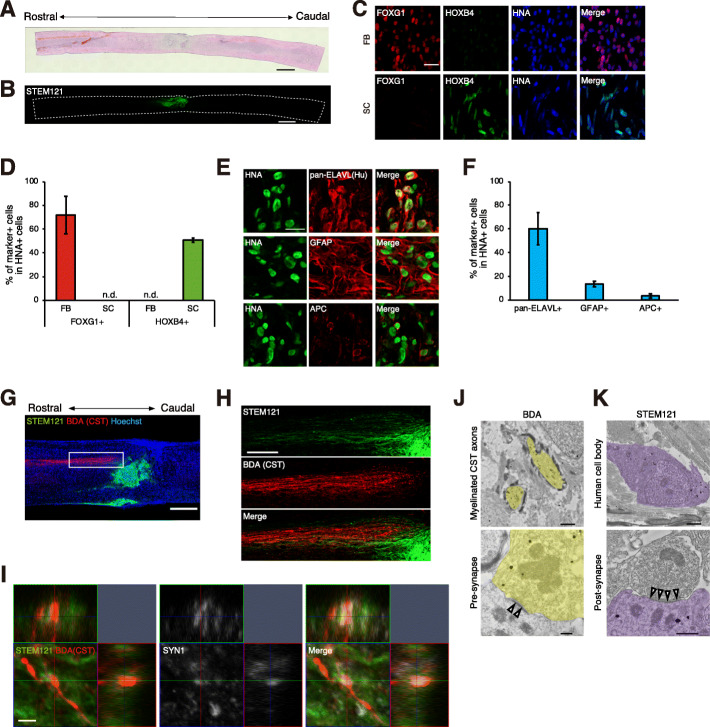
Transplantation of human iPSC-derived regionalized NPCs. **a** Representative image of the H&E-stained spinal cord of an SC-type NPC-grafted mouse at 9 weeks after injury. Scale bar, 1000 μm. **b** Representative immunohistochemical image of STEM121-positive cells in an SC-type graft at 9 weeks after injury. Scale bars, 1000 μm. **c** Immunohistochemical analysis of transplanted cells to detect regional markers. Regionalized NPCs, which were identified by the human-specific antibody HNA, retained their regional identities after transplantation. Scale bar, 20 μm. **d** Percentages of regional marker-positive cells among the HNA-positive transplanted cells at 9 weeks after injury (*n* = 3 mice; mean ± SD; n.d., not detected). **e** Immunohistochemical analysis of SC-type NPC transplanted cells to detect neuronal, astrocyte, and oligodendrocyte markers. Scale bar, 20 μm. **f** Percentages of marker-positive cells among the HNA-positive SC-type transplanted cells at 9 weeks after injury (*n* = 5 mice; mean ± SD). **g**, **h** Low (**g**) and high(**h**) magnification view of BDA-labeled CST axons with STEM121-positive SC-type transplanted cells. White box indicates the area shown in the high-magnification image. Scale bar, 500 μm (**g**) and 200 μm (**h**). **i** Confocal *z*-stack images showing synapse formation between CST axons and SC-type grafts. The upper squares show the *xz*-plane, and the right squares show the *yz*-plane. Scale bar, 2 μm. **j**, **k** Immuno-electron microscopic images of the CST-graft contact region. Immunogold-labeled BDA-positive myelinated CST fibers (J, *yellow*) and STEM121-positive graft cells (K, *purple*) were detected. These immunogold-labeled cells frequently formed pre- and post-synaptic structures. Arrowheads, postsynaptic density; scale bar, 1 μm (J, upper), 0.2 μm (J, lower), 1 μm (K, upper), 0.5 μm (K, lower)

We transplanted the regionalized NPCs into injured mouse spinal cords. At 9 weeks after transplantation, we confirmed that both FB-type and SC-type NPCs had been successfully engrafted onto injured spinal cords by immunostaining with the human-specific cytoplasmic marker STEM121 (Fig. 2a, b; Fig. S1A, B). As there are controversial reports on the maintenance of regional identity of transplanted NPCs [14, 27], we next examined the regional marker expression of grafted cells within the injured spinal cord. FB-type NPCs mainly expressed FOXG1, whereas HOXB4 was predominantly expressed by SC-type NPCs, suggesting that both FB-type and SC-type regionalized NPCs retained their regional identities after transplantation (Fig. 2c, d). The grafted cells mainly differentiated into ELAVL-positive neurons, and there was a small population of GFAP- or APC-positive glial cells, which was consistent with the in vitro data (Fig. 2e, f, Fig. S1C, D).

Next, we examined the interaction of the graft with the host spinal cord. BDA was injected into the primary motor cortex of host mice, and the corticospinal tract (CST) fibers were labeled. The SC-type graft extended neurites towards the host CST (Fig. 2g–i), but we could not find a connection between FB-type NPCs and the host CST (Fig. S1E, F). These CST-graft contact regions (shown by the white box in Fig. 2g) were further assessed by immuno-electron microscopy (iEM) analyses, and we frequently detected a number of functional pre- and post-synaptic structures labeled with BDA and STEM121, respectively (Fig. 2j, k). These iEM data were acquired from the restricted area of the adjacent section (white box in Fig. 2g) enriched in both CST fibers and grafted cells, and it is highly plausible that the host-derived CST fibers and the SC-type grafts were interconnected and probably contributed to synapse formation, although our gold-label iEM analyses are not direct evidence of synapse formation between CST fibers and the graft. Indeed, in the SC-type NPC graft, the presynaptic marker SYN1 colocalized with the bouton-like terminals of the CST and made contact with STEM121-positive grafted cells (Fig. 2i).

### Stratification of histological data identified specific subgroups that responded to SC-type NPC transplantation

**Fig. 3:**
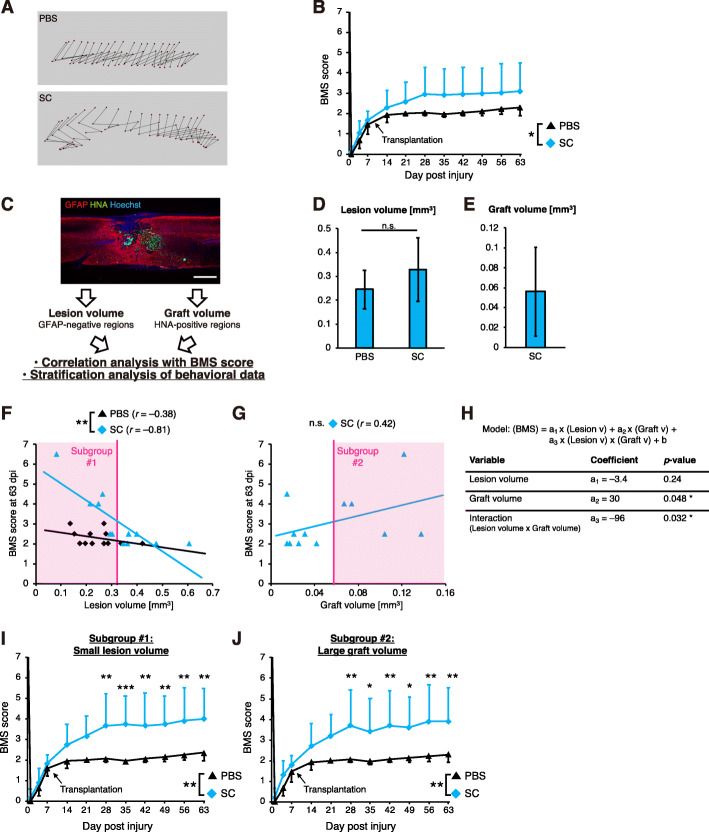
Motor function recovery in stratified subgroups. **a** Representative trajectories of hindlimb motions at 9 weeks after injury. **b** BMS scores of the mice transplanted with SC-type NPCs (the SC group) and the control mice (the PBS group) (SC group, *n* = 12; PBS group, *n* = 12; mean ± SD; *p < 0.05; two-way repeated measures ANOVA). **c** Histological parameter analysis. The lesion volume, graft volume, and spinal cord area at the lesion epicenter were assessed by the staining of sagittal sections of injured spinal cords with anti-GFAP and anti-HNA antibodies. Scale bar, 500 μm. **d**, **e** Quantification of the lesion volume (**d**) and the graft volume (**e**) (SC group, *n* = 12; PBS group, *n* = 12; mean ± SD; n.s., not significant; Student’s *t* test). **f**, **g** Correlation analysis of each parameter [the lesion volume (**g**) and the graft volume (**h**)] and the BMS score at 9 weeks after injury. The Pearson correlation coefficient *r* is shown. Statistical significance was assessed by regression models (**p < 0.01; n.s., not significant; multiple linear regression in F; single linear regression in G). The *k*-means-based unbiasedly selected subgroups for the stratification analysis are indicated as red boxes. **h** Multivariable regression analysis of BMS scores at 63 dpi in the grafted group (*p < 0.05). **i**, **j** BMS scores of selected cohorts according to the lesion volume (I) and the graft volume (**j**) (Subgroup #1: SC group, *n* = 6; PBS group, *n* = 10. Subgroup #2: SC group, *n* = 5; PBS group, *n* = 12 (the original cohort). mean ± SD; ***p < 0.001, **p < 0.01, *p < 0.05; two-way repeated measures ANOVA followed by a post hoc Tukey’s test. Subgroup #2 was compared to the PBS group in the original cohort

Next, we evaluated hindlimb locomotor function using the Basso Mouse Scale (BMS) [28]. In the group of animals transplanted with SC-type NPCs, the overall functional recovery was significantly greater than that in the PBS group (Fig. 3a, b), although FB-type NPC-grafted mice did not display functional improvement (Fig. S1G).

However, even in the SC-type NPC-grafted group, post hoc pairwise comparisons at each time point could not detect statistically significant differences in motor function. We found that some mice appeared to respond relatively well to transplantation; thus, we wished to exploit the phenotypic heterogeneity of the grafted mice to identify potential modifiers of motor function improvement resulting from SC-type NPC transplantation. For this purpose, we determined the histological parameters (the lesion volume and the graft volume of grafted mice and the lesion volume of ungrafted mice) (Fig. 3c–e) and investigated the interaction between these parameters and the BMS score (Fig. 3f, g). We confirmed that the lesion volume was comparable between controls and SC-type NPC-grafted mice (Fig. 3d). The lesion volume was well correlated with the BMS score in grafted mice, and we observed a statistically significant difference in the results of linear regression between the grafted and control groups, indicating that mice with small lesions had better responses to cell transplantation than those with large lesions (Fig. 3f). On the other hand, there was a relatively mild correlation between the graft volume and the BMS score (Fig. 3g).

We next examined the results for the multivariable regression model with interaction effects for the lesion volume and the graft volume (Fig. 3h). Using the dataset obtained from the grafted group, this model provided better fitting than univariable regression or simple multivariable regression (Table S1). The analysis of this model revealed that the graft volume had a positive effect on the BMS score, and the lesion volume negatively modulated this effect; that is, a larger engraftment led to better functional recovery, and this recovery was enhanced when the lesion was less severe.

We next divided the mice into subgroups (subgroups #1 and #2) by unbiased *k*-means clustering according to the histological data (Fig. 3f, g) and stratified the time-course datasets of the BMS scores according to these subgroups (Fig. 3i, j). In the subgroup of mice with small lesions (subgroup #1), significantly greater functional recovery was observed in grafted animals at 4 weeks post injury and was maintained better thereafter compared to that in control animals with similar lesions (Fig. 3i). We also observed a statistically significant improvement in mice with a greater number of grafted cells (subgroup #2) (Fig. 3j). These results indicate that SC-type NPC transplantation led to motor function improvement in substantial subsets of SCI mice.

**Fig. 4:**
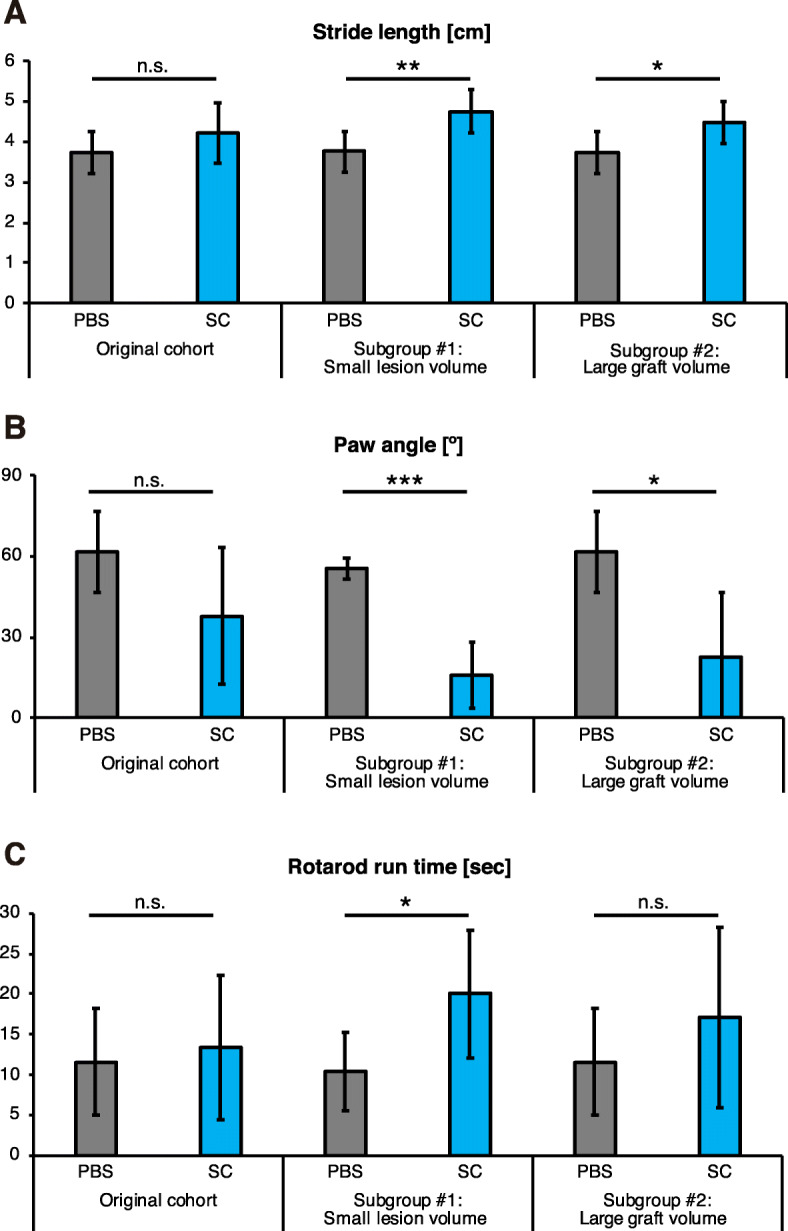
Behavioral improvement resulting from the transplantation of SC-type NPCs. **a** and **b** Stride length (**a**) and paw angle (**b**) according to the treadmill gait analysis (original cohort: SC group, n = 12; PBS group, *n* = 11. Subgroup #1: SC group, *n* = 6; PBS group, *n* = 9. Subgroup #2: SC group, *n* = 5. mean ± SD; ***p < 0.001, **p < 0.01, *p < 0.05; n.s., not significant; Mann-Whitney *U* test. Subgroup #2 was compared to the PBS group in the original cohort). **c** Time on the rotating rod in the rotarod test (original cohort: SC group, *n* = 12; PBS group, *n* = 12. Subgroup #1: SC group, *n* = 6; PBS group, *n* = 10. Subgroup #2: SC group, *n* = 5. mean ± SD; *p < 0.05; n.s., not significant; Mann-Whitney *U* test. Subgroup #2 was compared to the PBS group in the original cohort)

The same cohorts of grafted and control mice were also evaluated by behavioral analyses in addition to the assessment of the BMS score. In the treadmill gait analysis, we measured the stride length and the hindlimb paw angle (Fig. 4a, b). Although we could not detect any differences in the original cohort, NPC transplantation resulted in a significant increase in stride length and a significant reduction in the paw angle in subgroups #1 and #2. In the analysis of the rotarod test, the NPC-grafted mice in subgroup #1 remained on the rod significantly longer than the control mice (Fig. 4c). Therefore, we detected some statistically significant improvements in functional behaviors resulting from NPC engraftment in specific subsets of injured mice, which was consistent with the results of the BMS study (Fig. 3i, j). These data suggest that the lesion and graft volumes were important factors in NPC-induced motor function recovery and that SC-type NPCs promote functional restoration in mice with mild-to-moderate lesions and/or large grafts.

## Discussion

In the present study, we successfully differentiated human iPSCs into region-specific NPCs in vitro. When these NPCs were grafted onto the injured spinal cord in mice, they exhibited good engraftment properties, and their regional identities were retained. By comparing the engraftment effect of the two types of NPCs with different regional identities, we found that only the NPCs with a spinal cord identity promoted the functional recovery of SCI mice, highlighting the importance of the regional identity of cells used for engraftment in cell therapy for SCI.

Our culture method generated NPCs from human iPSCs after 12 days of differentiation, and these NPCs were capable of in vivo engraftment and supported motor function improvement in the injured spinal cord of mice. This time frame was much faster than that of conventional protocols developed for SCI treatment [3, 29]. The accelerated speed of this method is partially due to the pretreatment of cells with dual SMAD inhibitors and the hypoxic culture conditions, as previously demonstrated [30, 31]. While the rapid preparation of iPSC-derived NPCs is preferable, further characterization of such NPCs in terms of various aspects, such as genome stability and tumorigenesis, will be needed before moving forward with clinical trials.

While SC-type NPCs resulted in motor function recovery, FB-type NPCs failed to produce recovery. The difference of in vivo differentiation properties may account in part for the poor recovery by FB-type NPCs. In transplanted spinal cords, FB-type NPCs differentiated predominantly into neurons, whereas SC-type NPCs had more gliogenic competence. In addition, a previous work using human fetal NPCs reported that FB-type NPCs had the low graft integration efficiency [14]. Another study reported that the major difference between SC-type and FB-type NPCs was host circuit innervation; the host circuit can be interconnected with SC-type NPCs but not with FB-type NPCs [15]. Similar phenomena were also observed in our study. The FB-type NPCs formed dense clusters with poor neurite extension, and the host CST did not project into these cells (Fig. S1D, E). Notably, some SC-type grafts also projected to the ventral portion of the host spinal cord (Fig. 2g). Thus, SC-type grafts might interact with other descending tracts, in addition to CST. In fact, some studies revealed that graft-derived neurons could build interconnections with the host tracts in SCI animal models [5, 32, 33], and graft-host connectivity, at least in part, contributes to the therapeutic effects of NPC engraftment. Thus, the insufficient recovery resulting from FB-type NPC engraftment is partially due to the poor graft-host interconnection. However, in-depth characterization of the graft-host connection is needed to demonstrate the involvement of the connection in motor function recovery. Further analysis, including transsynaptic tracer labeling and functional intervention of the graft-host interconnection, will be beneficial.

We found that host responsiveness to NPC engraftment is linked to the lesion severity and graft size. This is also consistent with a previous report in which SC-type fetal NPC engraftment was shown to improve the motor function of SCI mice with mild lesions but not severe lesions [14]. The importance of lesion severity is also highlighted by the recent finding that treatment with anti-HMGB1 antibody reduced the lesion size of the injured spinal cord and improved motor function recovery synergistically with NPC transplantation [34]. To exploit the full potential of NPCs for SCI treatment, the use of such an additional approach to minimize the lesion size will be important.

In our protocols, Wnt and RA signaling were modulated to generate region-specific NPCs; on the other hand, fibroblast growth factors (FGFs) and sonic hedgehog (Shh) also play important roles in regionalization. Previous works reported that both RA and FGFs induce iPSC-derived NPCs with the spinal cord identity, but detailed characteristics are different; RA-treated NPCs have the anterior (brainstem-to-cervical) spinal cord identity, whereas FGF endowed NPCs with the more posterior identity (cervical and thoracic spinal cord) [35, 36]. Thus, our SC-type NPCs mainly corresponded to the anterior spinal cord, and it would be interesting to study the difference between the anterior and posterior spinal cord-specific NPCs in terms of functional recover of SCI.

On the other hand, Shh controls the dorsoventral patterning of NPCs. While our SC-type NPCs without Shh activation showed a dorsal identity [20], previous studies reported that iPSC-derived NPCs with a ventral spinal cord identity improved locomotor function in SCI animals [36, 37]. It was noted that the ventral spinal cord but not the dorsal cord is oligodendrogenic; thus, the effects of ventral SC-type NPCs might exceed those of dorsal SC-type NPCs through remyelination. However, a recent paper demonstrated that the regeneration of host axons in the injured spinal cord was more greatly enhanced by a dorsal SC-type fetal NPC graft than a ventral NPC graft [38]. Further study is crucial to clarify whether dorsal or ventral SC-type NPCs are suitable for SCI treatment.

In conclusion, we successfully generated human iPSC-derived NPCs with forebrain and spinal cord characteristics, and found that only the spinal cord-type cells enhanced the motor recovery of mice with spinal cord injury, especially in those with mild-to-moderate lesions. The present study highlighted the significance of the regional identity of iPSC-derived NPCs used for cell therapy for SCI.

## Methods

### Culture of undifferentiated iPSCs

The human iPSC lines 414C2 and 201B7 [39, 40] were cultured on mitomycin C-treated SNL murine fibroblast feeder cells in standard hESC medium (DMEM/F12, Sigma) containing 20% KnockOut serum (KSR) replacement (Life Technologies), 0.1 mM nonessential amino acids (Sigma), 0.1 mM 2-mercaptoethanol (Sigma), and 4 ng/ml fibroblast growth factor 2 (FGF2) (PeproTech) in an atmosphere containing 3% CO_2_.

### Generation of NPCs from iPSCs

Neural differentiation of iPSCs was performed as previously described [20, 21] with slight modifications. Briefly, iPSCs were pretreated for 6 days with 3 μM SB431542 (Tocris) and 150 nM LDN193189 (StemRD). They were then dissociated and seeded at a density of 10 cells/μl in media hormone mix (MHM) [41–43] containing selected growth factors and inhibitors in 4% O_2_/5% CO_2_. The growth factors and inhibitors included 20 ng/ml FGF2, 1x B27 supplement without vitamin A (Invitrogen), 2 μM SB431542, and 10 μM Y-27632 (Calbiochem). The day on which neural induction was started was defined as day 0, and the cells were reseeded at 50 cells/μl in MHM with 1x B27 and 10 μM Y-27632 on day 6. For forebrain-type NPC induction, 3 μM IWR-1e (Calbiochem) and 100 ng/ml FGF8 (Peprotech) were added from day 0–6 and from day 6–12, respectively. For spinal cord-type NPC induction, 3 μM CHIR99021 (Stemgent) and 1 μM retinoic acid (Sigma) were added on days 0–6 and on days 0–12, respectively, and 20 ng/ml FGF2 was added on days 6–12. For the control NPC induction, 20 ng/ml FGF2 was added on days 6–12. Cell viability on day 12 was assessed by trypan blue staining. For the transplantation experiments, 10 μM the ɣ-secretase inhibitor DAPT (Sigma) was administered on days 11–12 to suppress the tumorigenesis effect [29].

### In vitro neural differentiation analysis

On day 12, NPCs were plated *en bloc* on coverslips coated with polyornithine and laminin and induced to differentiate in 5% CO_2_ for 12 days. The medium was replaced with MHM supplemented with 1× B27 and 1 μM DAPT.

### SCI model and NPC transplantation

Female 8-week-old immunodeficient NOD/SCID mice were anesthetized with an intraperitoneal injection of ketamine (100 mg/kg) and xylazine (10 mg/kg), and a contusive SCI was produced at Th10 using an IH impactor (70 kdyn; Precision Systems and Instrumentation). Nine days after the injury, 5 × 10^5^/2 μl NPCs were injected into the lesion epicenter using a 5 μl Hamilton syringe with a metal needle at a rate of 1 μl/minute by a stereotaxic microinjector (Muromachi Kikai). An equal volume of PBS was injected into the lesion site in the vehicle control mice. We evaluated total 43 mice (16 SC-grafted, 15 FB-grafted, and 12 PBS-injected), and analyzed 33 mice (12 SC-grafted, 9 FB-grafted, and 12 PBS-injected) for behavioral and histological assays. Ten mice (4 SC-grafted and 6 FB-grafted) were excluded in this study, because grafts were not observed or distributed only in the epidural regions. One mouse in the PBS group could not be evaluated for treadmill gait analysis, because of strong spasticity. All experiments were performed in accordance with the Guidelines for the Care and Use of Laboratory Animals of Keio University (Assurance No. 13020) and the Guide for the Care and Use of Laboratory Animals (NIH).

### Motor function analysis

Hindlimb motor function was evaluated for 9 weeks after injury using the Basso Mouse Scale for Locomotion (BMS) [28]. We measured the stride length and the hindlimb paw angle of the animals when they walked on the treadmill using the DigiGait System (Mouse Specifics) at 9 weeks after injury. Motor function was also evaluated with a rotarod test, which utilizes a rotating rod apparatus (Muromachi Kikai) that consists of a plastic rod (3 cm diameter, 8 cm length) with a rough surface. At 9 weeks after injury, mice were plated on the rod while it was rotated at 20 rpm, and the amount of time the mice spent on the rod was measured.

### Kinematics analysis of locomotion

At 9 weeks after injury, the bilateral shoulders, hip joints, knee joints, ankle joints and toes of each animal were marked by colored markers, and the animals were recorded walking on the treadmill by 4 video cameras from the right anterior, right posterior, left anterior, and left posterior perspectives. The trajectories of these markers were analyzed by KinemaTracer software (KISSEI COMTEC).

### Anterograde labeling of the corticospinal tract (CST)

Biotinylated dextran amine (BDA; MW 10,000; 10% in DW, Invitrogen) was injected into the sensorimotor cortex at four sites (coordinates in mm B − 0.5 or − 1.5/L 0.5 or 1.5/D 0.7) to trace the descending CST fibers at 9 weeks after injury. The BDA tracer was stained by Alexa Fluor 555 conjugated to streptavidin, and histological analyses were performed.

### Immunohistochemistry and immunocytochemistry

All mice were deeply anesthetized and transcardially perfused with 4% paraformaldehyde (PFA) prepared in PBS at 9 weeks after injury. The dissected spinal cords were embedded in Optimal Cutting Temperature compound (Sakura) and sectioned in the sagittal plane at a thickness of 14 μm on a cryostat (Leica). In vitro*-*cultured cells were fixed with 4% PFA for 15 min at room temperature. The samples were stained with the following primary antibodies: anti-APC (mouse IgG2b, 1:300, Abcam, ab16794), anti-BRN3A (mouse IgG1, 1:500, Chemicon, MAB1585), anti-ChAT (goat IgG, 1:200, Chemicon, AB144P), anti-pan-ELAVL (Hu) (human IgG, 1:1000, a gift from Dr. Robert Darnell, Rockefeller University, New York, NY, USA), anti-FOXG1 (rabbit IgG, 1:500, Abcam, ab18259), anti-GFAP (rabbit IgG,1:1000, Proteintech, 16,825; rat IgG2a, 1:1000, Invitrogen, 13–0300), anti-HB9 (mouse IgG1,1:250, DSHB, 81.5C10), anti-HNA (mouse IgG, 1:200, Millipore, MAB1281), anti-HOXB4 (rat IgG2a, 1:100, DSHB, I12), anti-human cytoplasm (mouse IgG1, 1:100, STEM121, Takara Bio, Y40410), anti-ISL1/2 (mouse IgG2b, 1:1000, DSHB, 39.4d5), anti-SOX1 (goat IgG, 1:500, R&D, AF3389), anti-SYN1 (rabbit IgG, 1:2000, Sigma-Aldrich, S193-10UG) and anti-TUBB3 (mouse IgG, 1:300, Sigma-Aldrich, T8660). Nuclei were stained with Hoechst 33258 (10 μg/ml; Sigma-Aldrich). All samples were examined by using an LSM-710 confocal laser scanning microscope (Carl Zeiss) and a BZ-X710 fluorescence microscope (Keyence).

### Immuno-electron microscopy

The detailed procedure of pre-embedding immuno-electron microscopic analysis was described previously [44]. Briefly, the frozen sections of the spinal cord on the slide glasses were dried up with cool wind dryer and autoclaved for 1 h in pH 6.0 citrate buffer with the setting for 5 min at 105 °C, followed by the incubation with the blocking solution [5.0% Block Ace (DS Pharma Biomedical) solution with 0.01% saponin in 0.1 M PB] for an hour at 25 °C. For detecting BDA labeled CST fiber network, sections were incubated with Alexa Fluor 488 FluoroNanogold conjugated Streptavidin (SA-Ax488-NG, 1:100, Thermo Fisher Scientific) and mouse anti-human cytoplasm antibody (1:200, STEM121, Takara Bio, Y40410) in blocking solution for 72 h at 4 °C, followed by the incubation with Hoechst 33258 (1:1000, Sigma-Aldrich) and Alexa Fluor 555 conjugated goat anti-mouse secondary antibody (1:100, Thermo Fisher Scientific) for 24 h at 4 °C. For detecting human cells, sections were incubated with STEM121 antibody and Alexa Fluor 555 conjugated Streptavidin in blocking solution for 72 h at 4 °C, followed by the incubation with Hoechst 33258 and Alexa Fluor 488 FluoroNanogold conjugated goat anti-mouse secondary antibody (1:100, Thermo Fisher Scientific) for 24 h at 4 °C. The specific area of interest was identified by fluorescence microscope (LSM880, Carl Zeiss) where the transplanted human cells and BDA signals were colocalized. After 2.5% glutaraldehyde fixation in 0.1 M PB, nanogold signals were enhanced with silver enhancement solution for 10 min at 25 °C. Gold labeled sections were post-fixed with 1.0% Osmium Tetroxide (TAAB) for 2 h at 4 °C, *en bloc* stained with uranyl acetate for 20 min at 25 °C, and dehydrated through diluted ethanol (2 times of 50, 70, 80, 90, 100% EtOH for 5 min each, acetone for 5 min, 2 times of QY1 for 5 min, Epoxy resin with QY1 (1:1) for 1 h, 100% Epoxy resin (Oken) for 48 h at 4 °C and embedded into 100% Epoxy resin. After completing the polymerization for 72 h at 60 °C, resin block was trimmed and was ultrathin sectioned at 80 nm thickness with ultramicrotome (UC7, Leica). The ultrathin-sections were collected on the copper grids, stained with uranyl acetate and lead citrate. The sections were imaged with transmission electron microscopy (TEM; JEM-1400 plus, JEOL) at 100 keV.

### Quantitative RT-PCR

Total RNA was extracted from iPSC-derived NPCs on day 12 by using an RNeasy mini kit (QIAGEN) with DNase I treatment and reverse-transcribed with a ReverTraAce qPCR RT kit (Toyobo). qRT-PCR was carried out with SYBR Premix Ex TaqII (Takara Bio) on a ViiA 7 real-time PCR system (Applied Biosystems). The expression levels of each gene were normalized to that of *ACTB* using the comparative (ΔΔCt) method. The relative expression levels are presented as geometric mean ± geometric SD. The primer sets used in these experiments are as follows: *ACTB*, forward 5′-TGAAGTGTGACGTGGACATC-3′, reverse 5′-GGAGGAGCAA TGATCTTGAT-3′; *FOXG1*, forward 5′-CCCGTCAA TGACTTCGCAGA-3′, reverse 5′-GTCCCGTCGTAAAACTTGGC-3′; *EMX1*, forward 5′- AGGTGAAGGTGTGGTTCCAG-3′, reverse 5′- AGTCATTGGAGGTGACATCG-3′; *HOXB4*, forward 5′-ACGTGAGCACGGTAAACCCCAA-3′, reverse 5′- ATTCCTTCTCCAGCTCCAAGACCT-3′; *HOXC4*, forward 5′- TTCACGTTAGCACGGTGAAC-3′, reverse 5′- GACTTTGGTGTTGGGGAGTC-3′; *NESTIN*, forward 5′- TTCCCTCAGCTTTCAGGACCCCAA-3′, reverse 5′-AAGGCTGGCACAGGTGTCTCAA-3′.

### Stratification by lesion volume and graft volume

The lesions in the injured spinal cords were defined as GFAP-negative space that was compacted by the surrounding GFAP-positive reactive astrocytes [45]. The graft volume was approximated according to the detection of the HNA-positive regions. Three-dimensional analysis of the lesion/graft volume was performed as follows: four sagittal sections centered on the mid-sagittal axis that were separated from each other by 350 μm were immunostained with anti-GFAP and anti-HNA antibodies, and the GFAP-negative and HNA-positive areas were determined using ImageJ. The total lesion/graft volume, which was approximated as the total volume of the conical frustra, was calculated based on the following formula:

$$ V=\frac{h}{3}\times \left({A}_1+\sqrt{A_1{A}_2}+{A}_2\right), $$

where *A*_*1*_ and *A*_*2*_ are the areas of two consecutive sections, and *h* is the distance between them (350 μm).

To determine the subgroups for stratification, unbiased two-group clustering was performed using the *k*-means (*k* = 2) for the datasets for the lesion volume and graft volume.

### Statistical analysis

All data are presented as the mean ± SD. Statistical analyses were performed using Student’s *t* test for qRT-PCR and the immunostaining analyses, the Mann-Whitney *U* test for the behavioral analyses, two-way repeated measures ANOVA followed by a post hoc Tukey’s test for the BMS tests.

### Regression model

The BMS score at 9 weeks post injury was fitted using linear regression models with the histological parameters. The R-squared and Akaike information criterion (AIC) were used to evaluate the model fitting.

## Supplementary information

**Additional file 1: Supplemental Figure 1.** Characterization of FB- and SC-type NPCs. **Supplemental Figure 2.** FB-type NPC engraftment in SCI mice. **Supplemental Table 1.** Regression model of the BMS score with histological parameter

## Data Availability

All data generated or analyzed during this study are included in this published article.
