# Supplementary material for: Cell therapy for spinal cord injury by using human iPSC-derived region-specific neural progenitor cells
Source: Mol Brain. 2020 Sep 3;13:120. doi: 10.1186/s13041-020-00662-w (PMC7650268; doi:10.1186/s13041-020-00662-w)
Supplement: Supplementary file 1 — Additional file 1: Supplemental Figure 1. Characterization of FB- and SC-type NPCs. Supplemental Figure 2. FB-type NPC engraftment in SCI mice. Supplemental Table 1. Regression model of the BMS score with histological parameter [file 13041_2020_662_MOESM1_ESM.pdf]

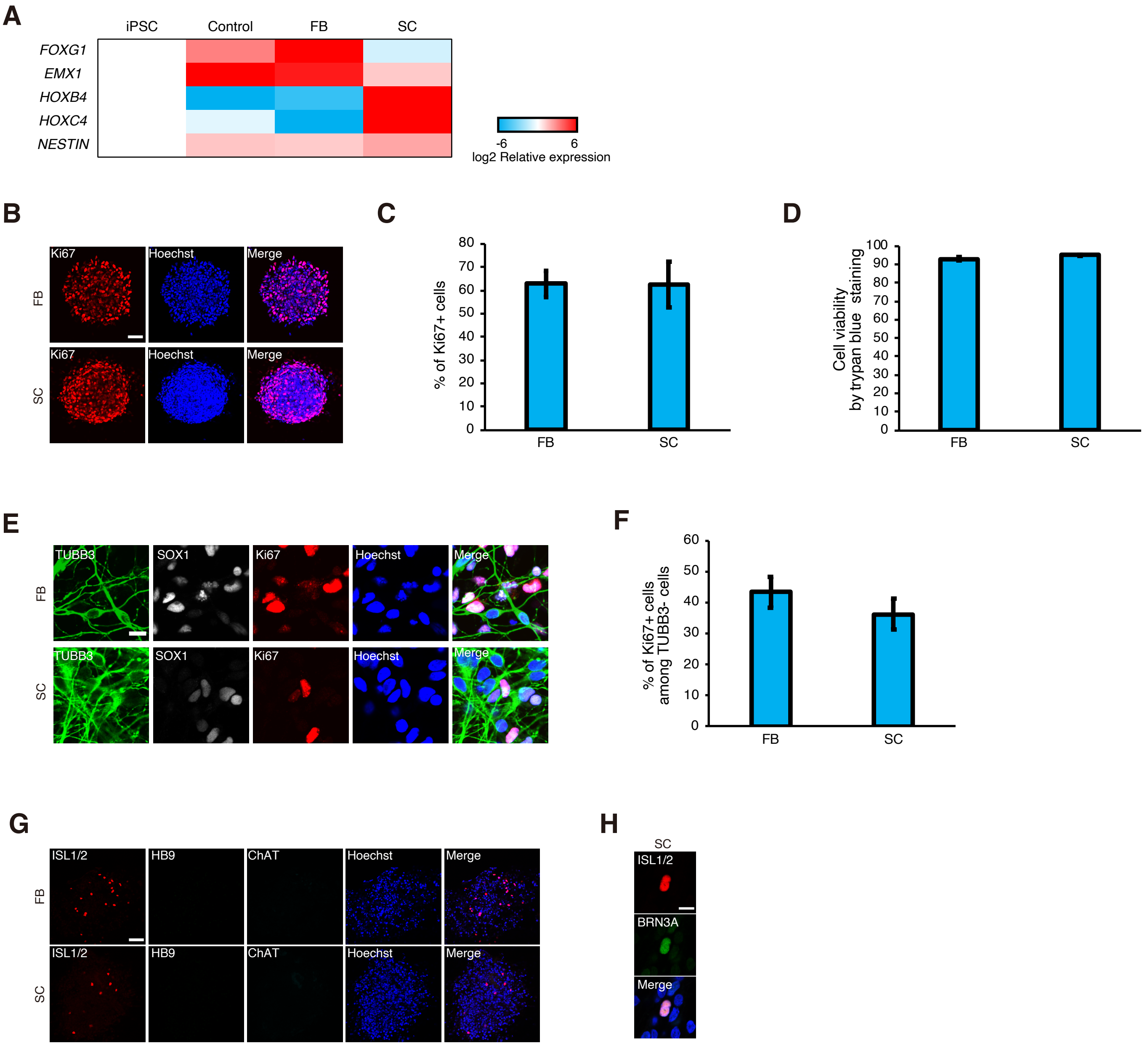

Supplemental Figure 1. Characterization of FB- and SC-type NPCs

(A) Heatmap summary of the qPCR analysis.

(B) Representative immunocytochemical images of Ki67-positive cells in NPC cultures. Scale bars, 50 µm.

(C) Quantification of the number of Ki67-positive cells (n = 3 independent experiments; mean ± SD).

(D) Quantification of cell viability by trypan blue staining (n = 3 independent experiments; mean ± SD).

(E) Immunocytochemical analysis of NPC-derived cells. Scale bar, 10 µm.

(F) Quantification of the number of Ki67-positive cells among TUBB3-negative cells in *in vitro* differentiation cultures (n = 3 independent experiments; mean ± SD).

(G, H) Immunocytochemical analysis of NPC-derived cells for neuronal subtype markers. Scale bar, 50 µm (G), 10 µm (H).

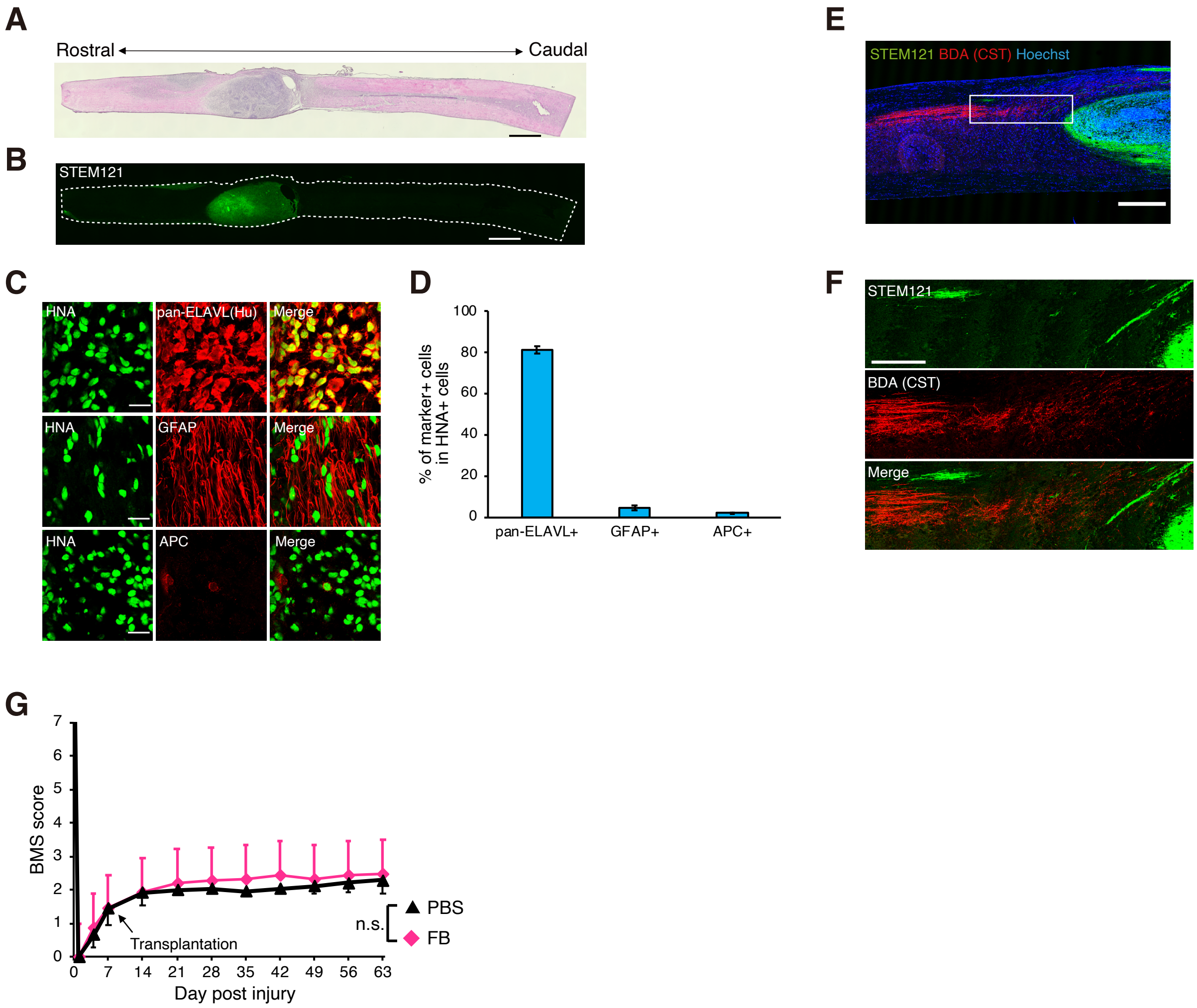

Supplemental Figure 2. FB-type NPC engraftment in SCI mice

(A) Representative image of the H&E-stained spinal cord of an FB-type NPC-grafted mouse at 9 weeks after injury. Scale bar, 1,000 μm.

(B) Representative immunohistochemical image of STEM121-positive cells in an FB-type graft at 9 weeks after injury. Scale bars, 1,000 μm.

(C) Immunohistochemical analysis of FB-type NPC transplanted cells to detect neuronal, astrocyte, and oligodendrocyte markers. Scale bar, 20 μm.

(D) Percentages of marker-positive cells among the HNA-positive FB-type transplanted cells at 9 weeks after injury (n = 3 mice; mean ± SD).

(E, F) Low (E) and high (F) magnification view of BDA-labeled CST axons with STEM121-positive FB-type transplanted cells. White box indicates the area shown in the high-magnification image. Scale bar, 500 μm (F), 200 μm (G).

(G) BMS scores of the mice transplanted with FB-type NPCs (the FB group) and the control mice (the PBS group) (FB group, n = 9; PBS group, n = 12; mean ± SD; n.s., not significant; two-way repeated measures ANOVA).

Supplemental Table 1. Regression model of the BMS score with histological parameter

Model	R-squared	AIC
Model 1. Univariable [Lesion volume] [a]	0.66	34.0
Model 2. Univariable [Graft volume] [b]	0.18	44.7
Model 3. Simple multivariable [c]	0.66	36.0
Model 4. Multivariable w/ interactions [d]	0.82	30.7

[a] (BMS) = a_1 x (Lesion v) + b
[b] (BMS) = a_2 x (Graft v) + b
[c] (BMS) = a_1 x (Lesion v) + a_2 x (Graft v) + b
[d] (BMS) = a_1 x (Lesion v) + a_2 x (Graft v) + a_3 x (Lesion v) x (Graft v) + b
